# Functional outcomes and mortality after multi-limb amputations following the 2023 Türkiye earthquake: a two-year follow-up study from a level I trauma center

**DOI:** 10.1186/s13018-025-06231-y

**Published:** 2025-08-30

**Authors:** Nihat Yiğit, İbrahim Alper Yavuz, Tahsin Aydın, Şahan Güven, Ceyhun Çağlar, İbrahim Bozkurt, Özgür Doğan, Enver Kılıç, Ahmet Özgür Yıldırım

**Affiliations:** 1 Department of Orthopedics and Traumatology, Sincan Training and Research Hospital, Ankara, Türkiye; 2 Department of Orthopedics and Traumatology, Bilkent City Hospital, Ankara, Türkiye

**Keywords:** Earthquake trauma, Multiple amputations, Crush injury, Disaster rehabilitation, Functional outcome, Sepsis, Prosthetic mobility

## Abstract

**Background:**

Multi-limb amputations are extremely rare and devastating injuries, particularly in the context of civilian disasters. The 2023 Türkiye earthquake resulted in a significant number of complex traumatic injuries, including multiple limb amputations. This study aims to investigate early mortality, surgical complications, and functional outcomes at two-year follow-up in civilian patients who underwent two or more limb amputations following the disaster.

**Methods:**

A retrospective analysis was conducted on 22 patients who sustained multiple limb amputations after the earthquake. Demographic data, amputation levels, surgical interventions, complications, and outcomes at two-year follow-up were recorded. The primary outcomes were mortality, the number and type of reoperations, and functional recovery at two years.

**Results:**

Among the cohort, two-limb amputations were performed in 17 patients, three-limb amputations in 4 patients, and four-limb amputation in 1 patient. Twenty patients required at least one additional surgical procedure during hospitalization, most commonly surgical debridement for soft tissue infection. Eight patients died, of whom five had undergone amputation of three or more limbs. Sepsis was the leading cause of death. Follow-up data at two years were available for 14 survivors. Of these, only 3 patients were able to ambulate independently using prosthetic devices. Phantom limb pain was reported in 6 patients, and all received medical management. The average hospital stay exceeded 80 days in patients with bilateral amputations.

**Conclusions:**

This study presents one of the largest case series of multi-limb amputees following a civilian earthquake. The findings highlight the high rate of reoperation, substantial mortality, and limited functional recovery at two-year follow-up in this population. Early multidisciplinary rehabilitation strategies should be prioritized to improve outcomes in future mass-casualty settings.

## Introduction

Natural disasters, particularly earthquakes, can lead to severe traumatic injuries and limb loss [1]. Earthquakes were estimated to be responsible for approximately 1.87 million deaths during the 20th century [2]. Previous studies have examined post-earthquake injury patterns and identified the leading causes of death as intracranial hemorrhage, fractures, dislocations, and crush injuries [3]. On February 6, 2023, at 04:17 local time, a 7.7-magnitude earthquake struck the Pazarcık district of Kahramanmaraş Province in Türkiye. Later the same day, at 13:24, a second earthquake with a magnitude of 7.6 occurred in the Elbistan district of the same province. These twin earthquakes affected an area spanning approximately 350 km and impacted 11 provinces, resulting in more than 46,000 deaths and over 110,000 injuries [4].

In disaster settings, limb amputation is often considered a life-saving intervention for individuals trapped under rubble, primarily to prevent life-threatening complications. Clinical conditions such as extensive tissue necrosis, vascular compromise, severe infection, or crush syndrome frequently necessitate amputation as the only viable treatment option [5, 6]. In major disasters such as the 1999 Marmara, 2005 Pakistan, 2008 Wenchuan, and 2023 Kahramanmaraş earthquakes, amputation has been reported as a necessary intervention for survival in many cases [7]. However, systematic and comprehensive studies on multiple limb amputations remain extremely limited in the current literature. In particular, data on follow-up outcomes such as prosthesis use, quality of life, and functional recovery after discharge are virtually nonexistent. The few existing publications are mostly limited to case reports and lack detailed information regarding amputation levels, associated complications, and follow-up outcomes. Furthermore, comprehensive analyses addressing early mortality and complication rates in this patient population are also absent. This gap represents a significant deficiency in disaster-related trauma management and rehabilitation planning.

As a Level 1 trauma center, our hospital admitted a large number of trauma patients simultaneously following the disaster. This study aims to evaluate the two-year outcomes, including postoperative complications, and mortality rates in patients who underwent simultaneous or near-simultaneous multiple limb amputations—an exceptionally rare clinical scenario. By analyzing the prognosis and complications observed in these cases, we intend to contribute valuable data to the global medical literature. To this end, detailed follow-up and clinical evaluations were conducted on patients admitted to our clinic after the earthquake.

## Materials and methods

This retrospective study aimed to evaluate the short-term clinical outcomes and two-year functional status of patients who underwent multiple limb amputations during their treatment within the Orthopedics and Traumatology Department following the earthquakes in Kahramanmaraş on February 6, 2023.Data collection and analysis commenced after obtaining approval from the Bilkent City Hospital Ethics Committee. The study included patients who met the following criteria: [1] were rescued from under rubble following the earthquakes on February 6, 2023; [2] underwent multiple limb amputations (≥ 2 limbs); [3] were hospitalized and treated at our institution; [4] had complete access to hospital records including surgical notes, nursing follow-up forms, Picture Archiving and Communication System (PACS) imaging, and discharge summaries; and [5] had at least two years of follow-up data available, including information on physical therapy, mobilization, and prosthesis use. The exclusion criteria were as follows: [1] patients who underwent amputation for non-earthquake-related causes (e.g., traffic accidents, industrial injuries); [2] patients with single-limb amputations; [3] patients with incomplete medical records (missing surgical notes, discharge summaries, PACS imaging, or follow-up data); [4] patients lost to follow-up; [5] patients who were not hospitalized at our center or were operated on elsewhere; [6] individuals with a prior history of limb deformity, prosthesis use, or amputation; [7] those with neurological conditions such as multiple sclerosis (MS) or cerebral palsy (CP), a history of stroke, or other motor dysfunctions that could affect post-amputation outcomes; [8] patients with severe cognitive impairments that precluded reliable data collection; [9] individuals with psychiatric conditions that prevented evaluation or communication; and [10] patients who underwent limb-sparing procedures such as fasciotomy but did not require amputation. As a result, a total of 22 patients who met al.l inclusion criteria were enrolled in the study. Demographic data (age, sex), length of hospital stay, number of surgical interventions, volume of blood products used (erythrocyte suspension, packed red blood cells; PRBC and fresh frozen plasma), postoperative complications (e.g., infection, sepsis, embolism, wound dehiscence), and associated traumatic findings (e.g., crush syndrome, pneumothorax, vertebral fractures, burns) were obtained from patient records. The length of hospital stay was calculated as the total number of days spent in the intensive care unit, orthopedic ward, and physical therapy unit.

### Perioperative period

All patients were admitted to our institution as part of emergency response efforts following the earthquakes centered in Kahramanmaraş on February 6, 2023, and were initially evaluated by a multidisciplinary team. This team consisted of specialists from orthopedic and trauma surgery, plastic surgery, general surgery, intensive care, anesthesiology, infectious diseases, radiology, physical medicine and rehabilitation, and psychiatry. All patients were initially assessed in the emergency department under a trauma protocol, focusing on resuscitation and hemodynamic stabilization. Following primary clinical examination and radiological evaluation by the orthopedic and trauma surgery team, advanced surgical planning was conducted based on the characteristics of the extremity injuries. Indications for amputation included progressive necrosis, closed compartment syndrome, crush injuries, vascular compromise (arterial or venous), major infections (e.g., phlegmon, gas gangrene), loss of functional integrity of the limb, and neurological disruption. Surgical indications were assessed using the Mangled Extremity Severity Score (MESS), a widely accepted classification system commonly referenced in the literature for guiding limb salvage decisions in traumatic extremity injuries. In our study, all patients who underwent amputation met the MESS criteria with a score of ≥ 7. This scoring system calculates a total score based on the type of injury, duration of ischemia, presence of shock, and the patient’s age [8, 9]. Surgical procedures were planned based on the patient’s overall clinical condition, as well as vascular and infection parameters. In cases where limb salvage was deemed feasible, limb-preserving surgical techniques—such as fasciotomy, debridement, or external fixation—were attempted. However, in the majority of cases, life-saving amputation was ultimately performed. Fasciotomies performed on the lower leg and forearm were specifically applied in cases presenting with compartment syndrome and ischemic muscle tissue. These interventions were conducted in accordance with the Clinical Practice Guidelines of the American Academy of Orthopaedic Surgeons (AAOS) and established Crush Injury Management protocols [8].

Amputation decisions were also made in collaboration with the departments of infectious diseases and plastic surgery. In cases involving osteomyelitis or gas gangrene, high-level amputation of the affected limb was planned in accordance with recommendations from existing literature. Kaur et al. (2022) reported that amputation was life-saving in a case of post-traumatic non-clostridial gas gangrene [10]. Kow et al. (2022) stated that in a case of humeral osteomyelitis following severe crush injury, amputation was performed prior to reconstruction to allow for debridement of infected tissue [11]. Apart from these cases, bilateral lower limb amputations were most commonly performed at the transfemoral (above-knee) level, while in upper limb cases, transhumeral and shoulder disarticulation levels were preferred.

### Surgical techniques

All amputations were performed under general or regional anesthesia using standard surgical protocols. The choice of amputation level—such as transfemoral, transtibial, transhumeral, or shoulder disarticulation—was determined based on the extent of soft tissue damage, vascular status, and presence of infection. Intraoperative debridement of devitalized tissue and microbiological culture sampling were routinely performed. In transfemoral amputations, primary closure was frequently achieved using posterior or skewed myocutaneous flaps, depending on tissue viability. In transtibial amputations, long posterior flaps or modified Burgess-type flaps were preferred to ensure optimal coverage and future prosthetic compatibility. Primary closure was attempted in clean, well-perfused wounds, while delayed closure or staged revisions were performed in cases with ongoing infection, ischemia, or poor soft tissue quality. All procedures were carried out by experienced orthopedic trauma surgeons, in collaboration with plastic and infectious disease specialists when necessary.

### Postoperative follow-up

Following surgery, patients were monitored either in the intensive care unit or the orthopedic ward based on their overall clinical condition. Patients requiring intensive care included those with complications such as polytrauma, crush syndrome, sepsis, or hemodynamic instability. Their management was conducted by a multidisciplinary team comprising specialists from orthopedics, intensive care, infectious diseases, plastic surgery, and nephrology. In patients transferred to the orthopedic ward, follow-up care included daily monitoring of vital signs, wound care, antithrombotic prophylaxis, fluid and electrolyte balance regulation, and antibiotic therapy. When necessary, laboratory tests—including C-reactive protein (CRP), procalcitonin, and complete blood count—as well as wound cultures were obtained to monitor and control infection. In cases with infected amputation stumps, secondary surgical interventions such as revision amputation or debridement were performed.

Pain management followed a multimodal analgesia approach throughout the perioperative and early postoperative periods. All patients received regular intravenous paracetamol (1 g every 6–8 h) combined with opioid-based analgesics such as tramadol (50–100 mg IV) or morphine (2–5 mg IV) as needed for moderate to severe pain. In patients experiencing refractory pain, patient-controlled analgesia (PCA) was employed during the first 72 h postoperatively. Non-steroidal anti-inflammatory drugs (NSAIDs) were avoided in patients with suspected crush syndrome or impaired renal function. Regional anesthesia techniques, such as nerve blocks, were not routinely used due to the emergency nature of the injuries and polytrauma presentations.

Postoperative complications such as surgical site infection, necrosis, embolism, deep vein thrombosis, pneumonia, and sepsis were observed. These events were documented in patient records and managed in coordination with the relevant medical specialties. Causes of early mortality were retrospectively assessed based on patient records and discharge summaries. Prior to discharge, all patients were evaluated by the physical medicine and rehabilitation department, and early mobilization protocols were initiated. Basic rehabilitation strategies—including sitting balance training, in-bed mobilization, and wheelchair use instruction—were implemented, particularly for patients who had undergone bilateral lower limb amputations. Extended follow-up was conducted through outpatient clinic visits and interviews after two years postoperatively. Structured questions were administered to patients regarding prosthesis use, physical therapy history, number of rehabilitation sessions received, daily mobility level, personal care abilities, and the presence of pain. Functional recovery was assessed qualitatively based on ambulation level, prosthetic use, and daily living activities. Although formal SIGAM scoring was not performed, patient categorization was guided by the SIGAM descriptive framework. Accordingly, patients were grouped as follows: [1] able to ambulate independently with a prosthesis (corresponding to SIGAM grades F–G) [2], requiring assistive devices in addition to a prosthesis (approx. SIGAM D–E), and [3] unable to ambulate (SIGAM A–C). This structured approach allowed for consistent evaluation across the cohort. These data were obtained from the hospital information system, PACS imaging archive, surgical notes, discharge summaries, and outpatient clinic interview records.

### Statistical analysis

All statistical analyses were performed using IBM SPSS Statistics version 25.0. Descriptive statistics were calculated as mean, standard deviation, median, and minimum–maximum for continuous variables, and as frequencies and percentages for categorical variables.

## Results

A total of 25 patients were initially enrolled in the study. Two patients were excluded upon their own request during the follow-up period, and one patient was excluded due to incomplete medical records. Consequently, 22 patients were included in the final analysis. The mean age was 30.0 ± 17.2 years (range: 4 to 67), with the majority of patients falling within the young adult (18–40 years) and middle-aged (40–65 years) groups. A total of 5 patients (22.7%) were under the age of 18. The gender distribution was balanced, with 11 male and 11 female patients. Detailed demographic and clinical information is summarized in Table 1.

Seventeen patients (77.3%) underwent two-limb amputations, four patients (18.2%) underwent three-limb amputations, and one patient (4.5%) underwent four-limb amputation. When classified by anatomic region, 9 patients had only lower limb amputations, 3 had only upper limb amputations, and 10 had both upper and lower limb involvement. The most frequently performed amputation levels included transfemoral, transtibial, and transhumeral, while shoulder and elbow disarticulations were less common. Multilevel amputations were observed in a significant number of patients, reflecting the severity of injury patterns (Table 1).

Surgical burden was substantial across the cohort. Patients with two-limb amputations underwent a mean of 5.1 surgical interventions, while those with three or more amputated limbs had a mean of 3.2 operations. Twenty of the 22 patients (90.9%) required at least one additional surgical procedure during hospitalization. The most common indication for reoperation was deep soft tissue infection at the amputation site (*n* = 7), followed by wound dehiscence (*n* = 6), sepsis requiring systemic control (*n* = 4), and non-infectious stump complications necessitating revision (*n* = 2). One patient experienced both sepsis and wound dehiscence concurrently, requiring multiple sequential interventions. No cases of slipped myodesis were identified during the two-year follow-up. All amputations were performed by experienced orthopedic trauma surgeons who employed appropriate myodesis or myoplasty techniques based on the anatomical level and soft tissue condition. Patients were regularly assessed for stump stability and prosthetic compatibility, and no signs of myodesis failure were documented. The detailed reoperation status for each patient is provided in Table 1.

Phantom limb pain emerged as a notable clinical issue during follow-up. Moderate to severe phantom pain within the first three months post-amputation was reported in 6 of 22 patients (27.3%). All affected individuals received pharmacologic treatment for pain management, including paracetamol, NSAIDs, and in selected cases, gabapentin or low-dose amitriptyline. Four patients who experienced severe phantom or residual limb pain were also referred for psychosocial and physical therapy interventions. Psychological approaches included supportive psychotherapy in all four cases, and Eye Movement Desensitization and Reprocessing (EMDR) therapy in two patients with post-traumatic stress symptoms. Physical therapy programs focused on stump desensitization, stretching, positioning, and progressive strengthening. In patients with lower limb amputations, pre-prosthetic training and gait education were initiated when medically feasible. None of the patients received mirror therapy due to logistical constraints. In addition to phantom limb pain, 6 patients reported residual limb pain localized to the stump region. Among these, 2 were clinically suspected to have neuroma-related pain based on localized tenderness and positive Tinel’s sign at the distal end of the stump. All patients with residual pain received oral pharmacologic therapy including NSAIDs, amitriptyline, or gabapentin. Physical therapy modalities such as stump desensitization, massage, and positioning techniques were applied in selected cases. No patients underwent surgical excision for neuroma.

Associated injuries were highly prevalent in this cohort. Crush syndrome was the most frequently documented trauma-related comorbidity, occurring in 10 patients (45.5%). Thoracic trauma, including pneumothorax (*n* = 6), hemothorax (*n* = 1), and bilateral pneumothorax (*n* = 1), was also frequently observed. Other trauma findings included a T12 compression fracture (*n* = 1), extensive second-degree burns (*n* = 1), and facial paralysis (*n* = 1). Only 3 patients had no documented associated injury.

Eight patients (36.4%) died during the early or intermediate postoperative period. Of these, five had undergone amputation of three or more limbs, indicating a potential correlation between amputation extent and mortality risk. The most common complication among deceased patients was sepsis, observed in 4 of the 8 fatalities. Mortality appeared to be clustered in patients with extensive amputations, systemic infections, and multiple associated injuries such as crush syndrome or thoracic trauma. The mortality status and corresponding clinical profiles of all patients are outlined in Table 1.

**Table 1:** Combined summary of patients with Multi-Limb amputation

Patient ID	Age	Sex(M/F)	Amputated Limbs	Amputation Levels	Cause of Reoperation	Phantom Pain	Associated injury	Mortality
1	28	F	4	Bilateral Transradial + Bilateral Transfemoral	Sepsis	No	Crush Syndrome	Yes
2	17	M	2	Bilateral Transtibial	Wound Dehiscence	Yes	T12 Compression Fracture	No
3	10	M	2	Right Transtibial + Left Transfemoral	Wound Dehiscence	No	Crush Syndrome	No
4	33	F	2	Right Transtibial + Left Syme	Wound Dehiscence	No	Hemothorax	No
5	18	M	2	Right Transtibial + Left Transfemoral	Infection (Soft Tissue)	Yes	Facial Paralysis	No
6	23	F	2	Bilateral Transtibial	Wound Dehiscence	No	Crush Syndrome	Yes
7	29	M	2	Bilateral Transtibial	Combined Complications (Sepsis + Dehiscence)	Yes	Second Degree Burn	No
8	67	M	2	Right Transhumeral + Right Transfemoral	Infection (Soft Tissue)	No	Crush Syndrome	No
9	47	M	2	Right Transtibial + Left Transfemoral	Infection (Soft Tissue)	No	Crush Syndrome	No
10	33	M	3	Left Shoulder Disarticulation + Right Transfemoral + Left Transtibial	Sepsis	No	Pneumothorax	Yes
11	27	F	2	Bilateral Transfemoral	Wound Dehiscence	Yes	Crush Syndrome	No
12	35	F	3	Right Transhumeral + Bilateral Transfemoral	Sepsis	No	Crush Syndrome	Yes
13	27	F	2	Bilateral Transtibial	Stump Revision (Non-infectious)	No	Bilateral Pneumothorax	No
14	44	F	2	Right Hip Disarticulation + Left Transfemoral	Sepsis	No	Pneumothorax	Yes
15	17	M	2	Right Transtibial + Left Transfemoral	Wound Dehiscence	Yes	Crush Syndrome	No
16	39	F	2	Bilateral Transtibial	None	No	No	No
17	36	F	2	Right Hip Disarticulation + Left Transfemoral	Infection (Soft Tissue)	No	Pneumothorax	Yes
18	4	M	2	Bilateral Transtibial	None	No	No	No
19	18	M	2	Right Transhumeral + Left Transtibial	Stump Revision (Non-infectious)	Yes	Pneumothorax	No
20	45	M	3	Bilateral Transtibial + Left Transradial	Infection (Soft Tissue)	No	Crush Syndrome	Yes
21	8	F	2	Bilateral Transhumeral	Infection (Soft Tissue)	No	No	No
22	44	F	3	Right Hip Disarticulation + Left Transtibial + Left Elbow Disarticulation	Infection (Soft Tissue)	No	Crush Syndrome	Yes

**Fig. 1:**
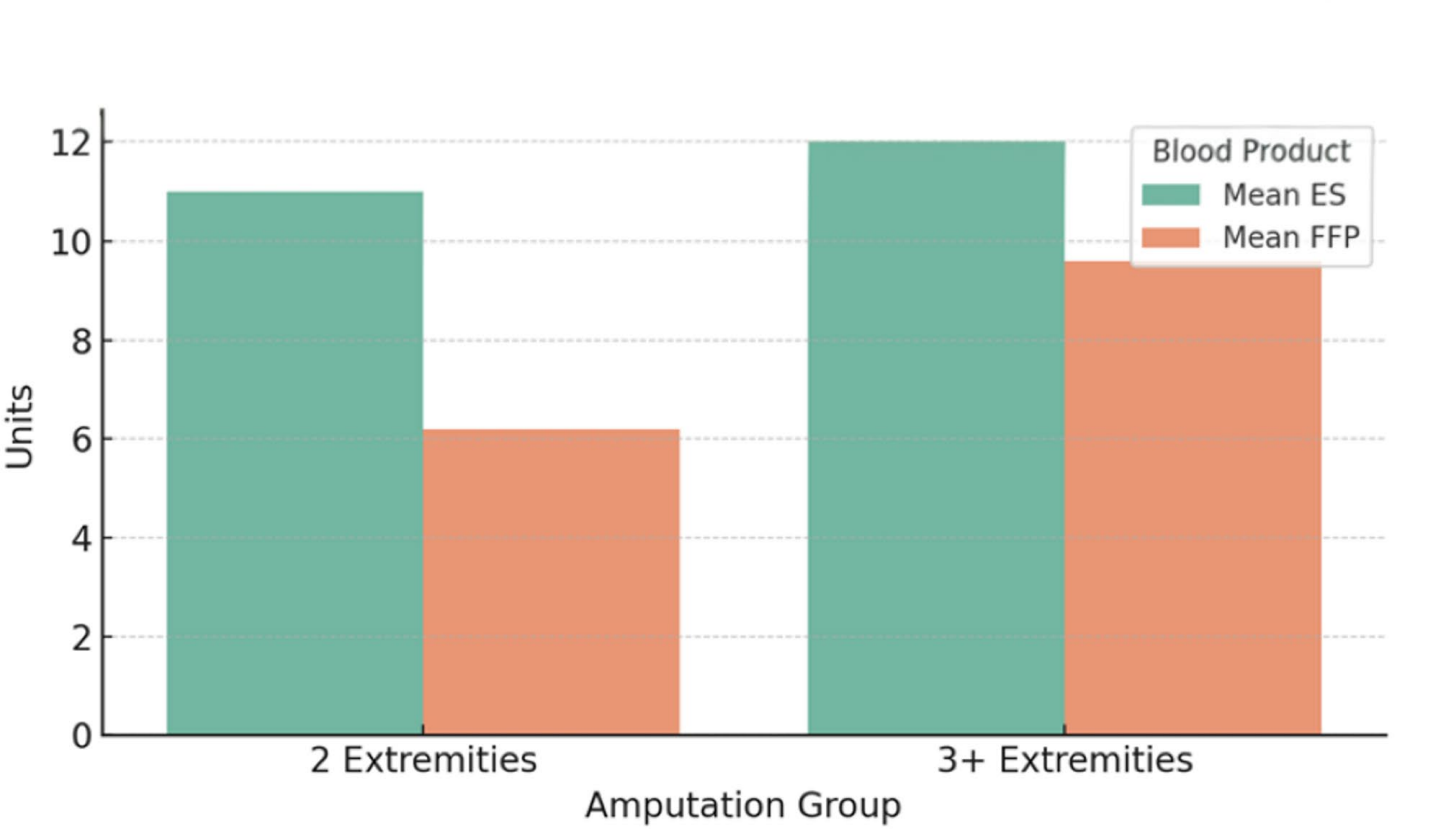
Comparison of mean erythrocyte suspension (ES) and fresh frozen plasma (FFP) units administered among patients with two versus three or more amputated extremities

In terms of blood product utilization, the most frequently administered component was erythrocyte suspension (ES). Among patients who underwent two-limb amputations, a mean of 11.0 units of ES and 6.2 units of fresh frozen plasma (FFP) were used. In patients with three or more limb amputations, the mean usage was 12.0 units of ES and 9.6 units of FFP (Fig. 1). These findings indicate a substantial transfusion requirement in both patient groups. Comparison of mean erythrocyte suspension (ES) and fresh frozen plasma (FFP) units administered among patients with two versus three or more amputated extremities.

Across the entire patient cohort, the mean hospital length of stay was 84.6 days. When grouped by number of amputated limbs, patients with two-limb amputations had a mean hospital stay of 98.1 days, whereas those with three or more limb amputations had a markedly shorter mean stay of 38.8 days.

Of the 14 surviving patients, 7 patients received physical therapy. Among these patients, the mean number of rehabilitation sessions was 64.3. Although all patients were fitted with at least one prosthesis, its effect on mobility level was observed to be limited. At the two-year follow-up, only 3 patients achieved independent ambulation using a prosthesis alone. The remaining 11 patients required additional assistive devices—such as walkers, crutches, or mobility aids—along with their prostheses for ambulation.

## Discussion

Multiple limb amputation is a severe clinical condition that is associated with high morbility and mortality rates [12, 13]. While most prior studies originate from military or conflict zones, the literature on simultaneous multiple amputations in civilian populations—particularly following natural disasters—remains sparse [14–16]. Our study presents the largest reported civilian case series evaluating medium-term outcomes following simultaneous multiple limb amputations. Furthermore, in terms of patient count, it constitutes the largest reported case series of simultaneous multiple amputations in the civilian population following a natural disaster. Among 22 patients, 90.9% required reoperation for postoperative complications, and 36.4% died. Only 21.4% of the surviving patients achieved unassisted ambulation with a prosthesis at the two-year follow-up, underscoring the immense functional burden faced by this population.

Demographic characteristics in our cohort are consistent with those reported in similar disaster settings. The mean age of 30.0 years and an equal male-to-female ratio align with earlier post-earthquake studies in Türkiye, Pakistan, and China. Notably, 22.7% of our patients were children, highlighting the susceptibility of pediatric populations during mass casualty events. Importantly, our study precisely documented the level and laterality of amputations for each patient, offering granular clinical data often missing from prior reports [17–19].

Lower extremity involvement was most common, observed in 40.9% of cases, consistent with the findings by Awais et al. and Bortolin et al., who identified below-knee and transfemoral amputations as the most prevalent after seismic events [5, 20]. In a study conducted by Aldaş et al. (2024) involving 356 pediatric patients, the amputation rate was reported as 2.8%. However, the distribution of amputations by upper or lower extremity and their anatomical levels were not specified [21]. In our study, all five pediatric patients were treated with primary surgery and underwent amputations involving two limbs. In 80% of the cases (*n* = 4), lower limb amputations—such as below-knee, above-knee, or transfemoral—were observed, whereas only one patient (20%) underwent bilateral upper limb (transhumeral) amputation. Additionally, the exact amputation level was clearly documented for each patient, making the level of detail in our study higher than that of existing reports in the literature.

Postoperative complications are common following multiple limb amputations, particularly in trauma-related cases. Doukas et al. reported complication rates nearing 80% in combat-related amputations, and Dillingham et al. identified infection and wound issues as leading causes for reoperations [12, 13]. Similarly, Delauche et al. found that 62% of patients required additional surgeries after the Haiti earthquake, mostly due to infections [14]. Phantom limb pain is another prevalent complication, affecting 30–85% of traumatic amputees, and is typically managed with pharmacological and psychological therapies rather than surgery [22, 23]. In our cohort, 90.9% of patients required at least one additional operation, with soft tissue infection (27.3%), wound dehiscence (22.7%), and sepsis (18.2%) being the most frequent indications. The mean number of surgeries was higher in two-limb amputees (5.1) than in those with three or more (3.2). Phantom limb pain occurred in 27.3% of patients, all of whom received medical management; two-thirds also participated in rehabilitation programs.

The presence of additional trauma is a well-recognized factor complicating amputation outcomes. Roy et al. reported that 42% of amputees after the Gujarat earthquake had associated injuries such as crush syndrome or spinal trauma [24]. Similarly, CDC data from the Haiti earthquake highlighted frequent concomitant complications, including pneumothorax and facial trauma [25]. High mortality rates are also consistently reported, particularly among patients with proximal amputations and sepsis. Qaarie et al. observed up to 40% one-year mortality after major lower limb amputations, while Delauche et al. reported a 35% mortality rate among multiple amputees post-Haiti earthquake, with sepsis being the leading cause [14, 26]. In our study, 8 of 22 patients (36.4%) died; among them, 62.5% had three or more limb amputations, and sepsis was the most common complication (also 62.5%). The most frequent comorbidity was crush syndrome (*n* = 10), followed by pneumothorax, T12 compression fractures, hemothorax, facial paralysis, and severe burns.

Substantial transfusion requirements have been frequently reported in disaster-related trauma cases. Koyuncu et al. noted that 60% of patients with earthquake-related crush syndrome required blood and plasma transfusions, while Vanholder et al. reported an average use of 6–9 units of erythrocyte suspension and 3–5 units of plasma during the Marmara earthquake [6, 27]. In a study conducted after the Boston bombing, it was reported that 80% of patients who underwent bilateral amputations required emergency blood transfusions [28]. In our study, the need for blood products was similarly high: two-limb amputees received a mean of 11.0 ES and 6.2 FFP units, while those with three or more amputations received 12.0 and 9.6 units, respectively. These results highlight the critical importance of transfusion support for maintaining hemodynamic stability and managing coagulopathy in post-disaster amputees.

Hospital length of stay following disaster-related amputations varies based on trauma severity, complications, and rehabilitation needs. Delauche et al. reported an average stay exceeding 90 days after the Haiti earthquake, largely due to complications and extended recovery [14]. Similarly, Asfuroğlu et al. observed hospitalizations over 60 days after the 2023 Türkiye earthquake [29]. In our cohort, the average stay was 84.6 days. Notably, patients with two-limb amputations had longer stays (98.1 days) than those with three or more (38.8 days). This inverse trend may reflect greater early mortality and physiological decline in the latter group.

Functional recovery after disaster-related amputation is shaped by access to early rehabilitation, prosthetic availability, psychosocial support, and general health. Knowlton et al. noted that delayed rehabilitation significantly limits independence [30]. Li-Tsang et al. reported only 18% of patients achieved independent ambulation postx-rehabilitation in the Ya’an earthquake [31]. Amatya and Khan (2023) reported that in groups receiving early physical therapy interventions, both pain control and mobilization outcomes were more successful, and long-term quality of life measures were significantly improved [32]. In our study, 50% of survivors engaged in physical therapy, averaging 64.3 sessions. While all received prostheses, only 21.4% (*n* = 3) achieved independent ambulation, and most (78.6%) required assistive support.

This study has several limitations. First, although the sample size is relatively small, it represents the largest reported series in the literature examining multiple limb amputations. Despite our institution being the largest Level 1 trauma center in the country and a major referral center for complex cases, the single-center and retrospective design limits generalizability, as the data reflect the patient profile of a specific facility. Lastly, as outcome data beyond the acute phase were only available for surviving patients, the results related to functional recovery and rehabilitation may not fully reflect the entire patient cohort. Although the study reports the sex distribution of the sample, no sex- or gender-based subgroup analyses were conducted. Therefore, potential differences in outcomes between sexes could not be evaluated and may be considered a limitation regarding the generalizability of the findings.

## Conclusion

In our study, patients who underwent multiple limb amputations exhibited high rates of mortality (36.4%), complication-related reoperations (90.9%), and limited long-term mobility. The findings indicate that this patient group is at high risk not only for surgical complications but also for prolonged physical dependency and functional impairment. Therefore, we recommend prioritizing early infection and sepsis control, implementing multidisciplinary complication management during hospitalization, and initiating individualized rehabilitation programs promptly after discharge in order to optimize recovery outcomes in this vulnerable population.

## Data Availability

The data that support the findings of this study are not publicly available due to institutional and patient privacy restrictions, but are available from the corresponding author upon reasonable request.
